# Effective and Novel Application of Hydrodynamic Voltammetry to the Study of Superoxide Radical Scavenging by Natural Phenolic Antioxidants

**DOI:** 10.3390/antiox8010014

**Published:** 2019-01-04

**Authors:** Stuart Belli, Miriam Rossi, Nora Molasky, Lauren Middleton, Charles Caldwell, Casey Bartow-McKenney, Michelle Duong, Jana Chiu, Elizabeth Gibbs, Allison Caldwell, Christopher Gahn, Francesco Caruso

**Affiliations:** 1 Department of Chemistry, Vassar College, Poughkeepsie, NY 12604, USA; nomolasky@vassar.edu (N.M.); lamiddleton@vassar.edu (L.M.); cc8caldwell@gmail.com (C.C.); cabartowmckenney@vassar.edu (C.B.-M.); michellemduong@gmail.com (M.D.); Jachiu@vassar.edu (J.C.); egibbs@vassar.edu (E.G.); alcaldwell@vassar.edu (A.C.); caruso@vassar.edu (F.C.); 2 Computing & Information Services, Vassar College, Poughkeepsie, NY 12604, USA; chgahn@vassar.edu

**Keywords:** antioxidants, flavonoid, superoxide, cyclic voltammetry, ROS

## Abstract

The reactions of antioxidants with superoxide radical were studied by cyclic voltammetry (CV)—and hydrodynamic voltammetry at a rotating ring-disk electrode (RRDE). In both methods, the superoxide is generated in solution from dissolved oxygen and then measured after being allowed to react with the antioxidant being studied. Both methods detected and measured the radical scavenging but the RRDE was able to give detailed insight into the antioxidant behavior. Three flavonoids, chrysin, quercetin and eriodictyol, were studied, their scavenging activity of superoxide was assessed and the molecular structure of each flavonoid was related to its scavenging capability. From our improved and novel RRDE method, we determine the ability of these 3 antioxidants to react with superoxide radical in a more quantitative manner than the classical CV. Density Functional Theory (DFT) and single crystal X-ray diffraction data provide structural information that assists in clarifying the scavenging molecular mechanism. Hydroxyls associated with the A ring, as found in chrysin, scavenge superoxide in a different manner than those found in the B ring of flavonoids, as those in quercetin and eriodictyol.

## 1. Introduction

Antioxidants are capable of preventing the oxidation of other molecules through the termination of detrimental free radical chain reactions by stabilizing the unpaired free electron characteristic of radical species. The superoxide radical (O_2_^−•^) is one of these free radicals that forms as a byproduct of aerobic respiration in biological systems. This radical can cause cellular damage, and is thought to contribute to aging as well as several degenerative diseases [1]. Biological systems are equipped with endogenous defenses against superoxide’s damaging effects, such as the enzyme superoxide dismutase [2] and catalase [3]. These defense mechanisms are assisted by exogenous antioxidants from dietary sources, mainly found in fruit and vegetables.

There are several sources which are used to generate the superoxide radical experimentally for its study [4], including an enzymatic reaction by xanthine dehydrogenase [5], or a non enzymatic option using phenazine methosulphate, NADH and molecular oxygen [6]. Once generated it is followed using spectrophotometric, colorimetric, chemiluminescence, and fluorescence detecting techniques. In addition, the superoxide can be trapped with 5.5-dimethyl-1-pyrroline-*N*-oxide (DMPO), and the resultant DMPO-OH adduct is detectable by ESR. These methods all *indirectly* measure superoxide concentration as they consist of measuring a product generated by superoxide consumption, and so adding an antioxidant will decrease the concentration of such product.

Additionally, superoxide can be generated by dissolving potassium superoxide, KO_2_, in a given solvent [7,8]. Since superoxide is affected by protons there are basically two variants for KO_2_ use, that is, operating in (1) an anhydrous solution, and (2) a strongly basic water environment [9]. A simpler method is to generate the superoxide radical in a voltaic cell, using classical cyclic voltammetry which detects decrease of current intensity of superoxide signal as a function of increasing concentration of antioxidants added to the voltaic cell [10]. Procedures used for generation and analysis of superoxide have been recently reviewed [11]; however, CV was not included. Our CV studies comprise (a) an accurate analysis of superoxide concentration when generated by KO_2_ through titration of anhydrous dimethylsulfoxide, (DMSO) solutions, (and we conclude that the KO_2_ generated superoxide radical is affected by instability); (b) the generation of superoxide directly in solution from dissolved molecular oxygen by cyclic voltammetry, and (c) an improved hydrodynamic voltammetry method using a rotating ring-disk electrode (RRDE) to analyze the efficiency of superoxide scavenging by 3 flavonoids, after generation of superoxide radical through bubbling O_2_ in an anhydrous DMSO solution.

Flavonoids are among the interesting natural compounds having antioxidant activity. Besides this activity, these compounds exhibit numerous advantageous biological properties, such as aids in digestive disorders [12]. The flavone skeleton structure provides ample substitution sites leading to a huge variety of structurally modified compounds. The subtle structural differences of flavonoids caused by hydroxyl and methoxy group substitution patterns in the three ring-system are known to affect their beneficial biological properties [13,14]. In this study we deal with three different flavonoids: quercetin, chrysin and eriodictyol, that are respectively, a flavonol, flavone and flavanone. Many traditional medicinal remedies throughout the world rely on citrus peels and eriodictyol is commonly found in citrus peels, along with related naringenin and hesperitin. Studying the molecular and crystal structure obtained through single-crystal diffraction methods allows us to investigate how these subtle structural changes can influence the biological properties. The crystal structure of eriodictyol was determined and the atomic coordinates employed to describe its reactivity with superoxide using Density Functional Theory (DFT).

It has been pointed out that the chemical information obtained through antioxidant research should be verified using several methods, particularly as the results are relevant to understand the oxidative deterioration of food and biological systems [4]. An important question [4] is: “What substrates are oxidized and what products are inhibited?” It is clear that to answer this question, a simple system for measuring antioxidant activity would be ideal to avoid potential side reactions. This is particularly true when studying flavonoids since it is known that they can sometimes act as pro-oxidants. Interestingly, the anti- or pro-oxidant behavior can be induced by the experimental conditions used to generate the radicals [15]. Also, structure-activity studies of natural phenolic antioxidants reveal the modes of inducing oxidation and the method used to determine oxidation greatly affect experimental results [4]. Therefore, our contribution, focused on using a method with significantly fewer variables in the experimental technique, the RRDE system, can provide valuable insight into antioxidant behavior.

## 2. Materials and Methods

### 2.1. Reagents

DMSO (anhydrous, ≥99.9%), tetrabutyl ammonium bromide (TBAB), [(2,2-dimethyl-6,6,7,7,8,8,8-heptafluoro-3,5-octanedionato) silver(I)], dibenzo-18-crown-6 ether and ethyl benzene were purchased from Sigma-Aldrich (St. Louis, Missouri, USA). DMSO was kept sealed in the original bottle and removed with a syringe in order to protect the solution from atmospheric moisture. Dibenzo-18-crown-6 ether and (TBAB) were kept dry in a desiccator over silica gel and used as received. Iodine, I_2_, (Sigma-Aldrich) was purified by sublimation before use. Potassium superoxide (Sigma-Aldrich) was kept in a desiccator and used as received. The antioxidants quercetin and chrysin, purchased from Sigma-Aldrich, and eriodictyol from Indofine Chemicals, Hillsborough, NJ, USA, were used as received.

### 2.2. Equipment

The potentiometric iodine titration was carried out in DMSO using an Orion pH/Ion meter with a platinum sensing electrode and nonaqueous Ag/AgCl reference electrode. The reference electrode was constructed with DMSO containing TBAB electrolyte in contact with a silver wire coated with AgCl. Cyclic voltammagrams were run using a Solartron SI 1287 Potentiostat/galvanostat (Solartron Analytical, Oakridge, TN, USA) controlled through Coreware© software. For the voltammetry study a three electrode configuration consisting of a gold disk (BASi) working electrode, a platinum wire counter electrode and the DMSO-based Ag/AgCl reference electrode was used.

Hydrodynamic Voltammetry was done at a rotating ring-disk electrode using the WaveDriver 20 bipotentiostat (Pine Research, Durham, NC, USA) with the MSR Electrode Rotator, from Pine Research Durham, NC, USA. The working electrodes, both the rotating ring and disk, were gold (Pine Research, Durham, NC, USA) with a coiled platinum wire counter electrode and a reference electrode consisting of a platinum wire immersed in 0.1 M TBAB in dry DMSO in a fritted glass tube. The electrodes were placed in a 5-neck electrochemical cell together with means for either bubbling or blanketing the solution with gas. Voltammograms were collected using Aftermath software provided by Pine Instruments. Careful cleaning of the electrodes was performed to clear potential film formation [16].

X-ray diffraction data were collected on a Bruker APEXII diffractometer (Bruker Corp. Madison, WI, USA) with MoKα radiation (0.71069 Å) at 125 K.

### 2.3. Procedures

#### 2.3.1. Potentiometric Titration of Superoxide with Iodine

KO_2_, crown ether, and TBAB were weighed and placed in a cell containing dry DMSO, platinum and reference electrodes. Aliquots of a known solution of iodine in ethylbenzene were added with the cell potential recorded after each addition. From the titration curve the initial concentration of superoxide in the solution could be calculated. The resulting concentration was always substantially less than that expected based on the mass of KO_2_ used in making the solution; the titration also demonstrated loss of superoxide over time. This result demonstrates the instability of the KO_2_ in DMSO and also loss of superoxide to residual water in the DMSO.

#### 2.3.2. Calibration of CV with Potassium Superoxide and Iodine

Due to the instability of KO_2_ in solution, the CV was calibrated through a titration of the superoxide with a standardized iodine solution. A 0.2 M iodine solution in ethylbenzene was made from purified iodine crystals. A stock potassium superoxide solution in DMSO was made by dissolving about 0.02 g KO_2_, 0.1 g dibenzo-18-crown-6 and 0.02 g tetrabutylammonium bromide in 25 mL anhydrous DMSO. Ten mL of the stock superoxide solution was placed in the electrochemical cell with the 3 electrodes and bubbled for 10 min with nitrogen gas. A voltammogram was recorded from −1.10 V to +0.50 V at 50 mV/sec showing the superoxide oxidation peak at −0.30 volts. An aliquot of iodine solution was added to the cell which was then stirred for 2 min, at which point another voltammogram was recorded. The iodine aliquots were continued until the superoxide oxidation peak had disappeared. The peak heights are measured from an extrapolated baseline, and plotted against the amount of iodine added. The oxidation peak disappears when the superoxide is completely removed by reaction with iodine and the titration curve breaks at this point allowing the calculation of the initial superoxide concentration of the stock solution. This titration curve can be recalculated in terms of oxidation peak height due to concentration of superoxide ion, yielding a calibration curve for the cyclic voltammetry (CV), in terms of bulk concentration of superoxide ion.

### 2.4. Superoxide Scavenging by Antioxidants

#### 2.4.1. CV Study

Stock solutions of quercetin and chrysin were made to approximately 7.9 × 10^−4^ M in anhydrous DMSO and used in trials. For the experiment, a solution of tetrabutylammonium bromide in anhydrous DMSO is bubbled for 10 min with a dry O_2_/N_2_ gas mixture to establish the dissolved oxygen level in the electrochemical cell. An initial blank is run on this solution by cycling the cell from a potential of +0.10 volts relative to the reference electrode, moving initially in the negative direction to a potential of −1.5 volts. The reduction peak was observed at −0.6 volts due to the reduction of molecular oxygen (O_2_) to the superoxide ion, O_2_^−•^, on reversal of the sweep, an oxidation peak is observed at −0.3 volts. The height of the oxidation peak is measured and together with the previous calibration gives the concentration of superoxide ion in the vicinity of the electrode surface. Next, an aliquot of one of the antioxidants was added, the solution bubbled with the gas mixture for 2 min and the CV rerecorded. Again the oxidation peak is measured and the change in magnitude from the “blank” is taken as the amount of superoxide removed by the scavenger. From the known addition of flavonoid and the amount of superoxide ion removed, the ratio of moles of superoxide scavenged to moles of flavonoid added may be calculated. Additional aliquots of each flavonoid are added to generate a reaction curve for each flavonoid.

#### 2.4.2. RRDE Study

The voltaic cell was prepared with 0.1 M TBAB in 50 mL of DMSO. The cell was purged with nitrogen gas (1 atm) for five min and then oxygen gas (1 atm) for ten min. The Au/Au electrode was then rotated at 500 rpm while the disk was swept from 0.1V to −1.5V and the ring was held constant at −0.25 V, the sweep rate was held constant at 25 mV/s. All tests were made with chrysin, quercetin and eriodictyol solutions at approximately 7.9 × 10^−4^ M in anhydrous DMSO.

### 2.5. Computational Study

Calculations were performed using software programs from Biovia, San Diego, CA, USA. Density functional theory code DMol^3^ was applied to calculate energy, geometry and frequencies implemented in Materials Studio 7.0 (PC platform) [17]. We employed the double numerical polarized (DNP) basis set that includes all the occupied atomic orbitals plus a second set of valence atomic orbitals, and polarized d-valence orbitals [18], and correlation generalized gradient approximation (GGA) was applied including Becke exchange [19] plus Perdew correlation [20] (PBE). All electrons were treated explicitly and the real space cutoff of 5 Å was imposed for numerical integration of the Hamiltonian matrix elements. Solvent effects were included and results show that water and DMSO have similar geometrical effects. The self-consistent-field convergence criterion was set to the root-mean-square change in the electronic density to be less than 10^−6^ electron/Å^3^. The convergence criteria applied during geometry optimization were 2.72 10^−4^ eV for energy and 0.054 eV/Å for force.

### 2.6. Diffraction Study

A suitable single crystal of eriodictyol grown in a 1:1 ethanol/water solution was chosen for single crystal X-ray diffraction studies. Data were collected at 125 K; details are given in Table 1. The molecular structure was solved using Bruker-SHELXTL software [21] with refinement of full-matrix least-squares on F^2^; data collection and refinement parameters are summarized in Table 1.

Data have been deposited at CCDC code 1829519 and are available upon request, https://www.ccdc.cam.ac.uk.

## 3. Results

### 3.1. Generation and Measurement of Superoxide Radical by Cyclic and Hydrodynamic Voltammetry

#### 3.1.1. Cyclic Voltammetry

**Figure 1:**
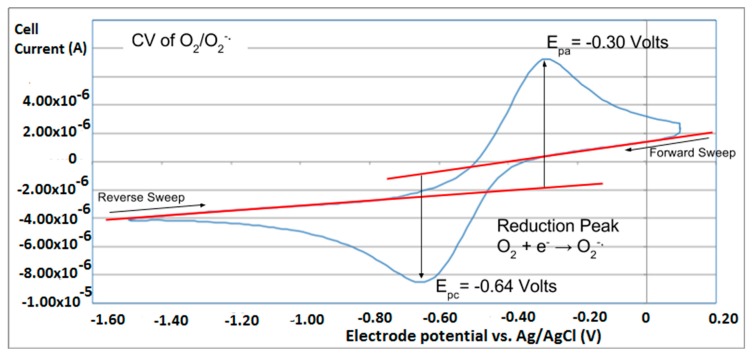
Cyclic voltammogram of dioxygen/superoxide redox couple.

In this study, we use the forward sweep of cyclic voltammetry (CV) to generate the superoxide radical ion and subsequently, in the reverse sweep, we measure the amount of the superoxide radical ion remaining (Figure 1). Upon addition of the antioxidant the CV is modified and the difference is due to the amount of superoxide radical ion scavenged by the antioxidant. The superoxide radical ion is stable over the time of the experiment in an anhydrous aprotic solvent containing no reactive species so the loss of superoxide radical ion is attributed only to the added antioxidant. In this method, the “forward” sweep drives the working electrode negative, creating the superoxide radical ion:

O_2_ + e^−^ → O_2_^−•^ reduction of oxygen to superoxide radical

Subsequently, the superoxide radical ion generated at the working electrode can react with reactive species in the solution near the electrode surface. On the “reverse” scan, the remaining superoxide radical ion gives up its electron to the electrode in an anodic reaction. The electrode reaction in both the forward and reverse directions is measured as a current seen as peaks in the CV:

The height of each peak is proportional to the quantity of superoxide generated and oxidized and so changes in peak height will inform us about the reactivity of the antioxidant studied.

As shown in Figure 1, the reduction-oxidation is reversible. The peak height is related to bulk concentration through the Randles-Sevcik equation:

I_p_ = 2.686 × 10^5^ n^3/2^ A c D^1/2^ v^1/2^ @25 °C

where n is the moles of electrons transferred, A is the electrode area, c is the concentration, D is the diffusion coefficient and v is the scan rate. In the present work we report the calibration of the method with solutions of superoxide from the reagent KO_2_, using this equation:

I_p_ = m [O_2_^−•^]

where m is the slope of the calibration curve. The KO_2_ solution was titrated with standardized iodine using the oxidation peak in the CV.

#### 3.1.2. Hydrodynamic Voltammetry (RRDE)

**Figure 2:**
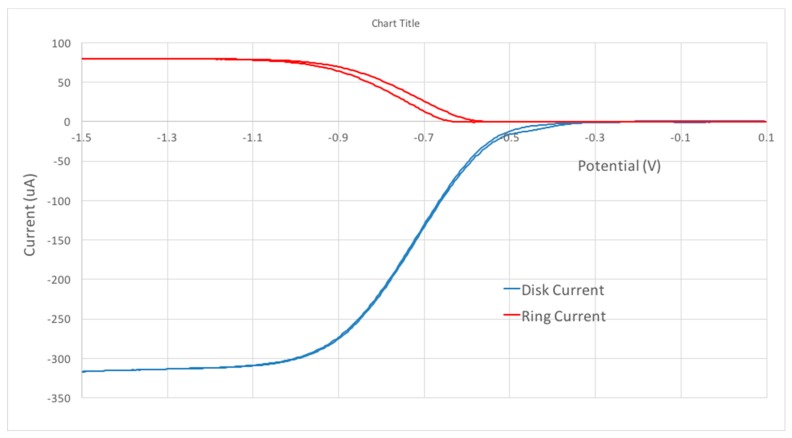
The cathodic, bottom, and anodic, top, currents from the oxygen reduction to superoxide at the disk and subsequent oxidation back to oxygen at the ring. Solution is 0.1 M TBAB in dry DMSO, saturated with 1 atm O_2_.

Hydrodynamic voltammetry at a rotating ring-disk electrode (HV-RRDE or just RRDE) was applied to the study of the antioxidant reaction with superoxide radical ion. RRDE uses two independent working electrodes, a central disk electrode with an encircling ring electrode. The rotation of the electrode sets up movement of the solution so that solution is brought up to the disk and then swept across the face of the electrode past the ring. The electrical potential of the disk can be made sufficiently negative that dissolved molecular oxygen is reduced to the superoxide radical ion and subsequently transported to the ring electrode which is held at a sufficiently positive potential that the superoxide radial is oxidized back to molecular oxygen. The method allows the monitoring of radical scavenging by the antioxidant through measurement of collection efficiency, N, which is simply the ratio of the ring to the disk current. This allows a straightforward interpretation of the antioxidant activity. The RRDE thus measures two currents (Figure 2). The process at the disk electrode is the reduction of oxygen to superoxide radical (O_2_ + e^−^ → O_2_^−•^), while the reverse reaction occurs at the ring electrode (O_2_^−•^ → O_2_ + e^−^). We focus on the latter reaction (Figure 2, top). The experiment starts with the disk electrode at 0.10V and the ring set to −0.25V. The disk potential is then swept in the negative direction; when the disk electrode has sufficient negative potential to generate superoxide, a cathodic current is observed as shown in Figure 2, bottom. The outward flow then sweeps the solution towards the ring electrode (fixed potential −0.25 V), allowing the superoxide reverse reaction yielding the anodic current (Figure 2, top).

### 3.2. Potentiometric Superoxide Titration with Iodine

The reagent KO_2_ is unstable and cannot be obtained in sufficient purity to make solutions of known concentration. In addition, any water present in the DMSO will react with the superoxide, further decreasing the amount in solution. The concentration of superoxide can be determined by titration with iodine:

2KO_2_ + I_2_ = O_2_ + 2KI potentiometric titration with Ag or Pt electrode

**Figure 3:**
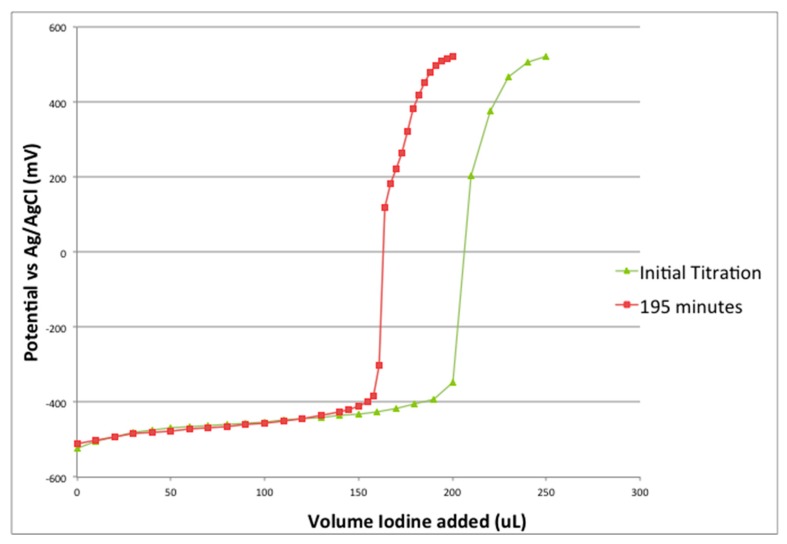
Potentiometric titration of KO_2_ with iodine, I_2_.

Titration of potassium superoxide solutions typically showed only 50–70% of expected superoxide concentration and instability over time. The superoxide concentration decreased 5% in 75 min and 24% over 195 min (Figure 3). Because of the instability of the superoxide standards, we have used a successive elimination type calibration where the superoxide concentration of the standard solution is successively reduced by the addition of known amounts of iodine, in effect we have repeated the titration using CV as the detection method.

### 3.3. CV Calibration

**Figure 4:**
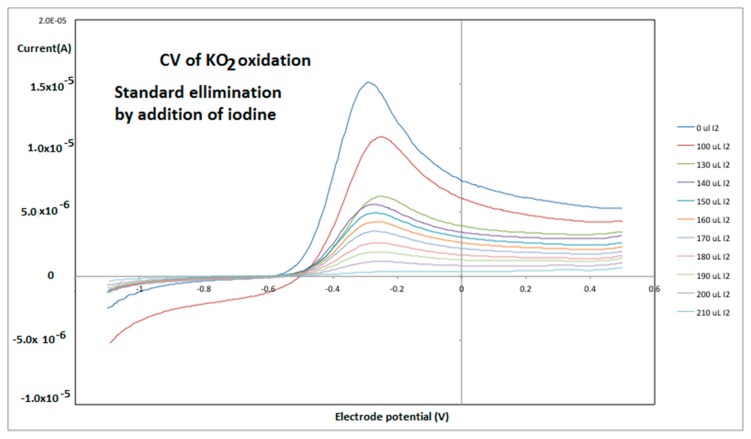
Voltammogram of KO_2_ in DMSO with additions of iodine in ethylbenzene. The electrode potential was swept from −1.1 volts vs the non-aqueous Ag/AgCl reference to +0.5 volts at 50 mV/s. After each iodine addition the cell solution was bubbled with the dry O_2_/N_2_ for 2 min.

A 0.1 M iodine solution was prepared from iodine previously purified through sublimation in dry ethylbenzene. A solution of KO_2_ in dry DMSO was titrated with aliquots of the iodine solution recording a cyclic voltammogram for the solution after each step in the titration (Figure 4). The loss of peak anodic current, as read by the CV, was then correlated with the loss of superoxide radical that had reacted with the added iodine.

**Figure 5:**
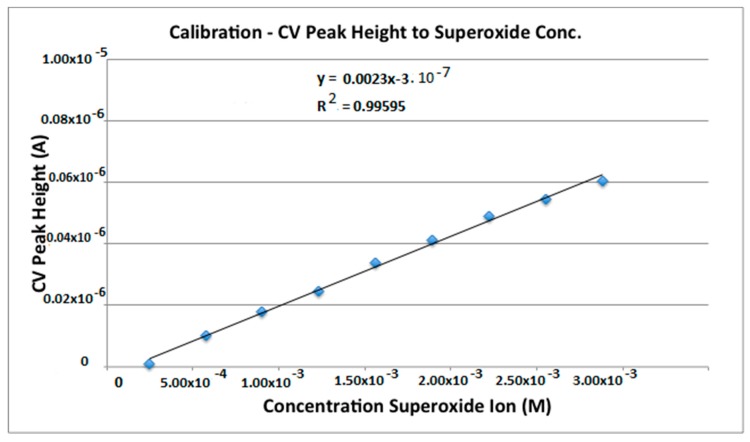
Calibration curve for the oxidation peak of superoxide in DMSO.

The endpoint of the titration was used to calculate the initial concentration of superoxide in the stock KO_2_ solution. Next, the amount of superoxide in solution for each of the voltammograms was calculated and the titration curve restated as a calibration curve for the voltammogram as shown in Figure 5. Finally, a linear fit of the plot was performed and an equation obtained.

### 3.4. Measuring Antioxidant Activity

**Figure 6:**
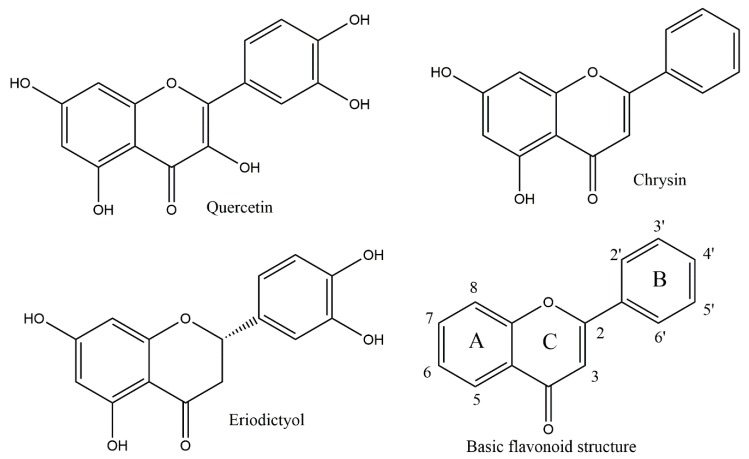
Flavonoids studied.

**Figure 7:**
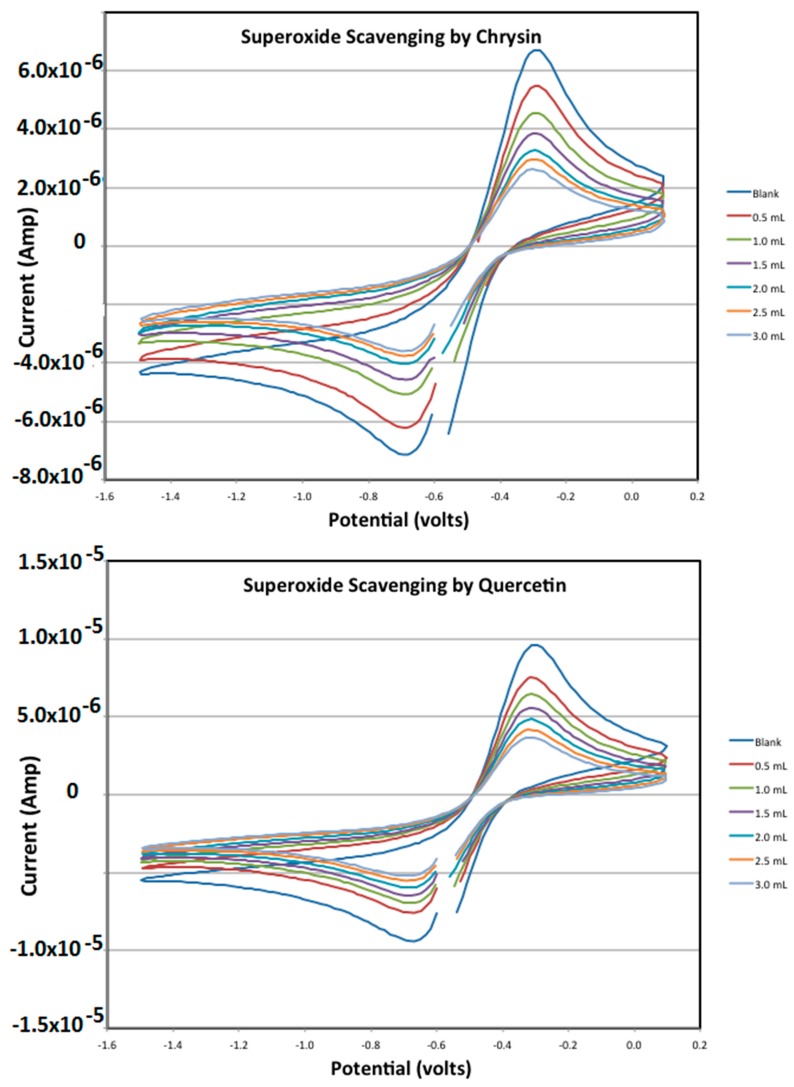
CV (cyclic voltammetry) of chrysin and quercetin showing the decrease in oxidation peaks as their aliquots were added.

For compounds tested (Figure 6), an initial CV experiment without added antioxidants (blank) gives the amount of superoxide produced by the forward scan that is available to react later with the scavengers. The antioxidants cause a decrease in the oxidation peak height indicating a loss of superoxide radical ion as shown in Figure 7. That is, the reaction in this process represents a typical scavenging process for a flavonoid (f–OH) species, by a radical (R^•^) as superoxide (f–OH + R^•^ → f–O^•^ + RH).

**Figure 8:**
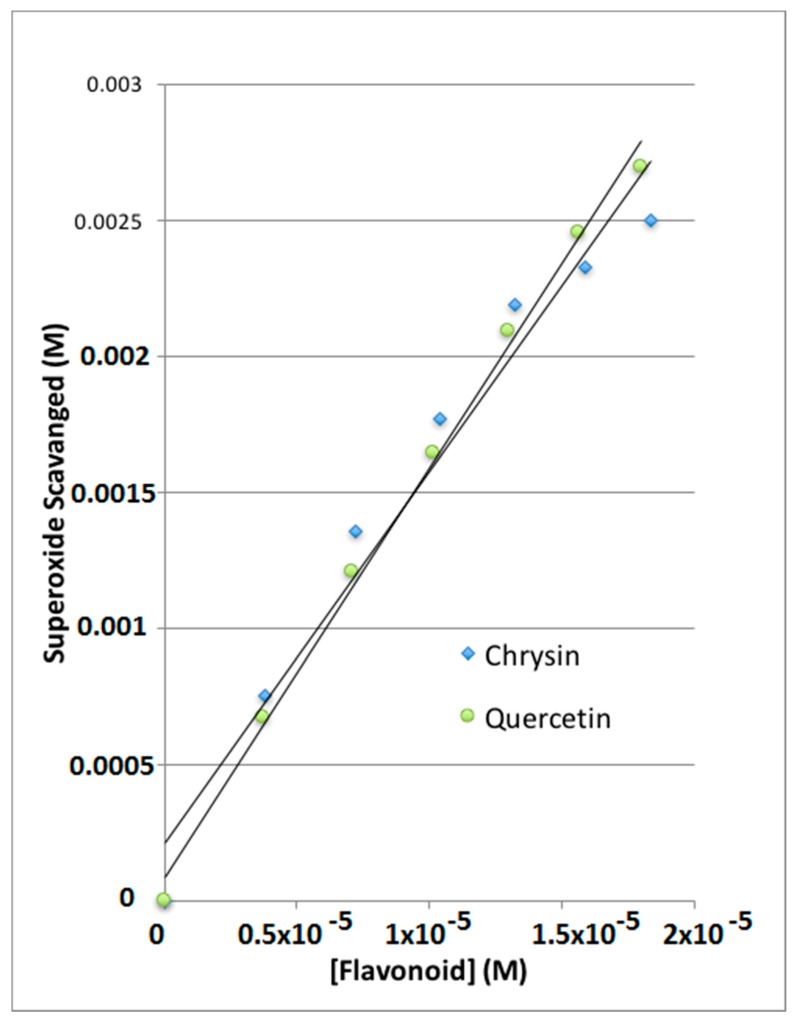
Superoxide scavenging by chrysin and quercetin.

The amount of superoxide radical scavenged is calculated as the difference in oxidation peak height from the initial blank run using the calibration equation to convert the signal from amps to molar concentration of superoxide. A plot of this (Figure 8) shows the similar response of each of the flavonoids. The calibration allows the direct comparison of moles of superoxide scavenged per mole of flavonoid added to the solution.

### 3.5. Diffraction

**Figure 9:**
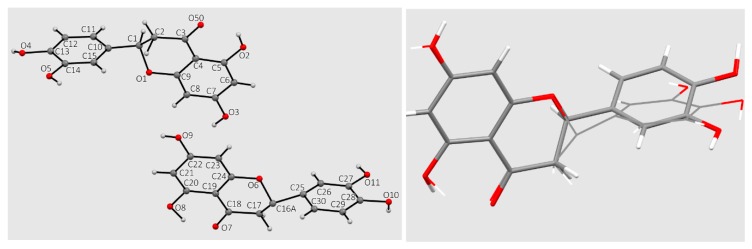
Atom labeling for non-H atoms in the 2 independent molecules. For clarity, disordered C atoms C1 and C16A not shown, left. Superposition of the 2 independent molecules of eriodictyol in the asymmetric unit, right. One molecule is in heavier stick mode while the second is narrower stick mode.

Several eriodictyol crystals were investigated because there was uncertainty in space group assignment, possibly due to the presence of a pseudo center of symmetry. This is related to disorder and different conformations in some of the phenyl ring atoms and at the chiral carbon. A variety of refinement situations were attempted and one possibility led to Z = 2 in acentric monoclinic space group *Pc*. However, a pseudo-inversion center is almost certainly present and so the centric *P2_1_*/*c* space group could be considered with Z = 1. Ultimately, refinement in *Pc* led to an *R*-value of about 5% whereas for refinement in *P2_1_*/*c*, the R-value was about 15%. So we continued our analysis with the acentric space group *Pc*. Thus, the crystal contains a racemic mixture in the solid state since both enantiomers appear in a disordered arrangement (a disordered solid-solid solution). Since the stereoisomers are comparable in overall shape and the hydrogen-bonding intermolecular interactions are similar, formation of a disordered solid solution is not surprising. The crystal structure and its inverse were refined, and the *R*-values were similar. There is evident disorder in the chiral carbon atoms of the two eriodictyol molecules over two sites (corresponding to the two enantiomers) at approximately 50:50 occupancy. The atom labeling and a superposition of the two molecules in the asymmetric unit of *Pc* is shown in Figure 9. Apart from the disorder at the chiral carbon, the two molecules differ from each other in the orientation of the phenyl ring resulting from rotation about the chiral carbon bond.

**Figure 10:**
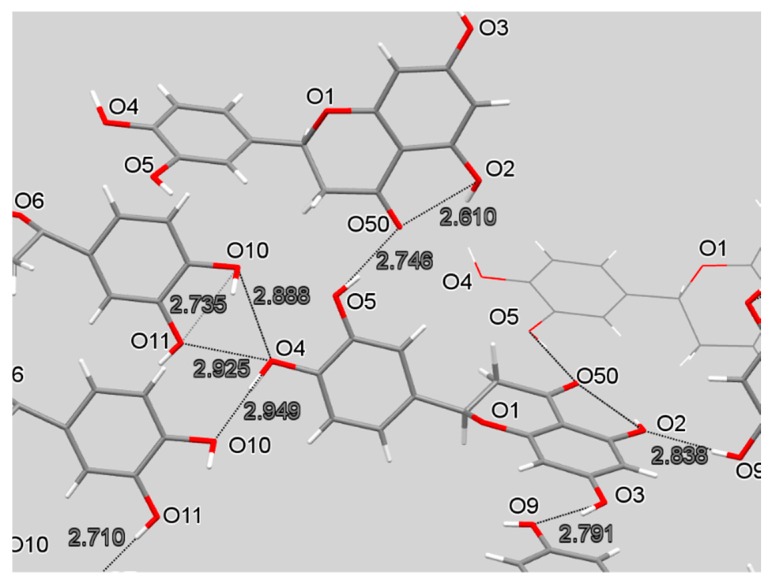
H-bond network of eriodictyol.

There are numerous hydrogen bonds (eight) in the crystal structure leading to a pattern composed of an infinite array of short interactions; it is shown in Figure 10. A related pattern was described earlier for hesperitin [22] and naringenin [23]. Although the hydrogen bond pattern seen in these molecules shares common features with eriodictyol (this work), none have as widespread and three-dimensional a pattern as eriodictyol. Of those flavanones whose crystallographic structures are available in the Cambridge Structural Database (CSD), hesperitin is most similar to the functional group substitution pattern found in eriodictyol—it only differs by substitution of an ortho methoxy group for a hydroxyl on the attached phenyl ring. However, eriodictyol’s two molecules both have the angle between the phenyl ring and the carbonyl-containing ring at close to 77° while for hesperitin, the planes of the phenyl ring and the carbonyl-containing ring are almost co-planar, about 3°.

## 4. Discussion

### 4.1. Cyclic Voltammetry

As seen in the Results section, chrysin and quercetin are effective scavengers of superoxide, and their slope can indicate the proficiency of antioxidant scavenging, Figure 8. Yet we can guess some difficulty in accurately quantifying this scavenging action, for instance when comparing more antioxidants, if we want to know which antioxidant is stronger. The uncertainty arises from the fact that measuring distances from the red tangent lines of Figure 1 to the peak maxima can be subjective, also when studying the lines obtained after adding the aliquots. For this reason, a more accurate method would be recommendable. This is provided by the RRDE technique, where the measurement of data is specifically obtained at a given potential at the ring electrode.

### 4.2. RRDE

**Figure 11:**
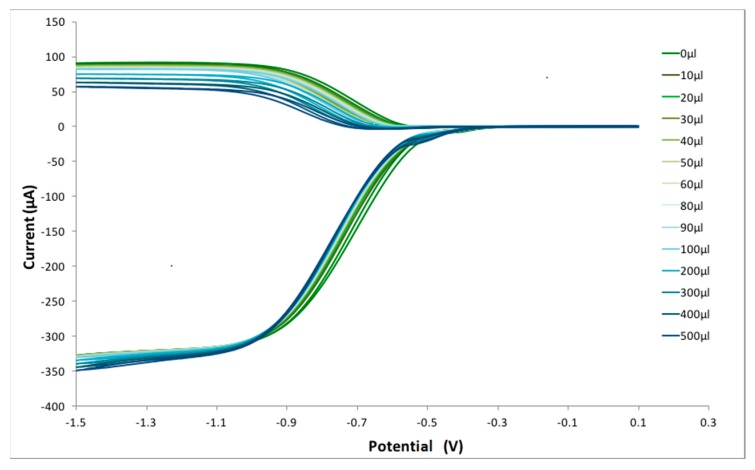
Voltammogram showing the effect of eriodictyol on the superoxide redox reaction for each added aliquot.

**Figure 12:**
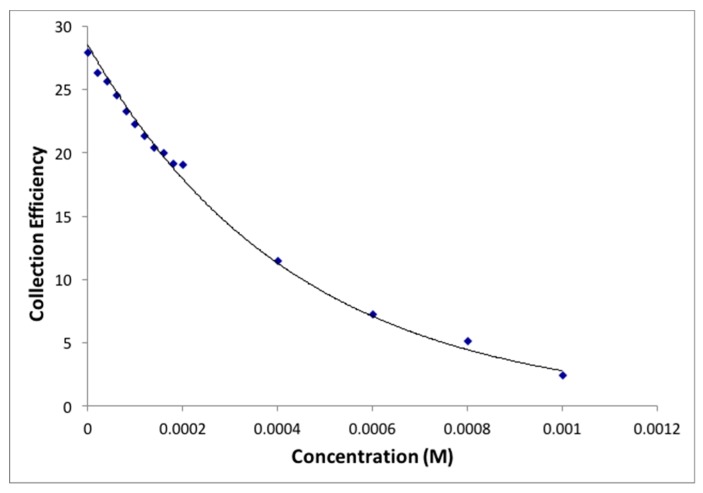
Collection efficiency for quercetin.

**Figure 13:**
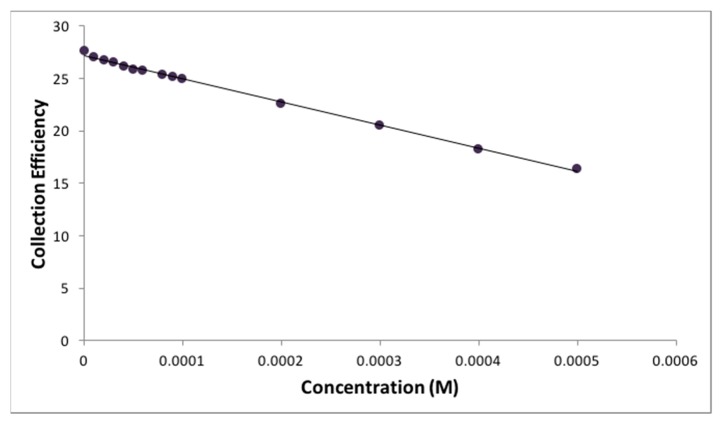
Collection efficiency for eriodictyol.

**Figure 14:**
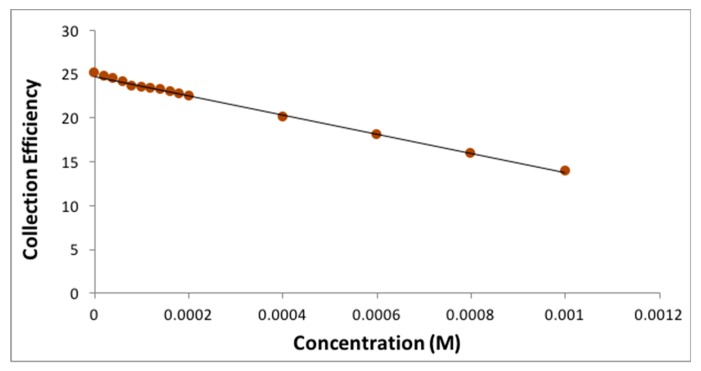
Collection efficiency for chrysin.

The collection efficiency is obtained from the voltammogram, and Figure 11 shows this amount for eriodictyol, by dividing the I value at the ring and that at the disk for each added aliquot, against the concentration of flavonoid in the solution. Figure 12, Figure 13 and Figure 14 show the results for quercetin, eriodictyol and chrysin and confirm the trend shown in the classical CV study: the more antioxidant added, the less superoxide radical detected, in this case at the ring electrode. The different slopes are indicators of scavenging capabilities for each flavonoid; these are included in Table 2, which also shows the ratio between hydroxyl groups for quercetin/eriodictyol/chrysin (5:4:2). The ratio between eriodictyol and chrysin slopes (4:2) is exactly the same as their hydroxyl ratio, and indeed, the number and location of hydroxyl groups are believed to affect the scavenging ability of antioxidants [14]. In contrast, quercetin activity seems to be higher, e.g., the slope ratio of quercetin/eriodictyol/chrysin = 9.6:4:2 differs from the hydroxyl ratio 5:4:2, Table 2. It is not clear why quercetin scavenges many more superoxide radicals in comparison with eriodictyol and chrysin, nor the reason of the non linear behavior of quercetin in Figure 12, which differs markedly from Figure 13 and Figure 14. Since quercetin possesses the larger number of hydroxyls it may be that a quercetin-specific behavior between partially dehydroxylated quercetin radicals is at work, such as the fusion of 2 radicals generating new species and thus producing the non linear portion of Figure 12. The fact that the excessive scavenging activity of quercetin occurs at low concentration is also intriguing. We conclude that further particulars need to be considered in the RRDE experiment dealing with superoxide radical and antioxidants, including for instance, convection and diffusion phenomena.

From this study it is seen that the rotating ring disk electrode technique, RRDE, provides more quantitative results for scavenging antioxidants when compared with standard CV methods. Indeed, we confirm the utility and specificity of such a technique, already applied in studying the superoxide radical in battery investigations using a Au/Au electrode with a platinum reference electrode and a platinum counter electrode [24].

### 4.3. X-ray Diffraction

The X-ray structure of eriodictyol provided the atomic coordinates used as a starting point for DFT calculations to study the molecular mechanism responsible for superoxide scavenging. The two molecules in the asymmetric unit have disordered chiral carbon (C2) and show extensive H-bonding throughout the crystal structure.

### 4.4. DFT Study

**Figure 15:**
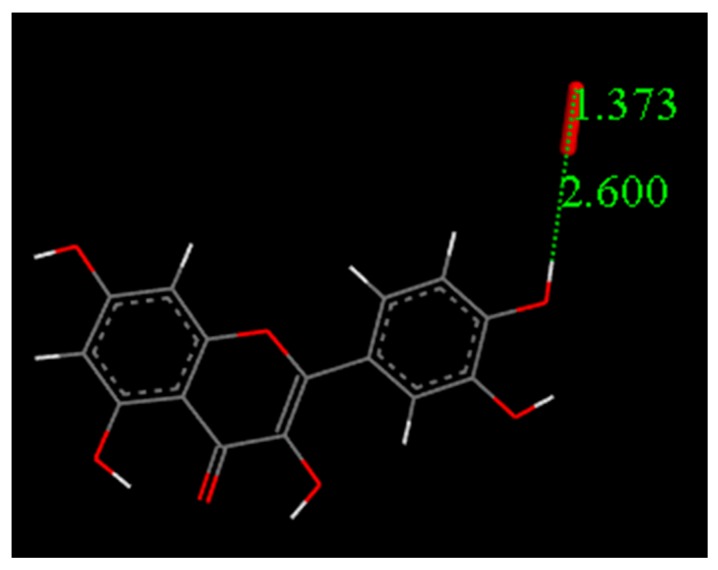
DFT Initial State for scavenging superoxide radical by quercetin. Both reagents are separated by van der Waals parameters, 2.60 Å.

**Figure 16:**
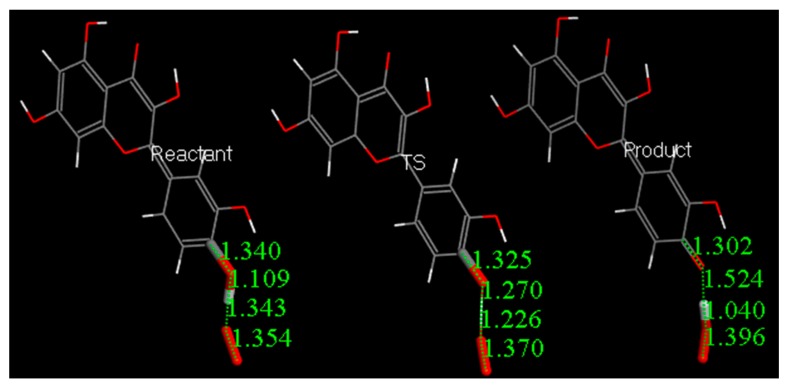
DFT scavenging of superoxide by quercetin. Minimized Reactant, transition state (TS) and Product.

Atomic coordinates from the quercetin crystal structure [25] were used as the starting point in calculations to analyze its scavenging activity towards the superoxide radical. After minimizing the geometry of quercetin and O_2_^−•^, both molecules were posed at van der Waals separation, 2.60 Å, for the extraction of H^•^ in position 4′ (Figure 15). For this initial state of reactivity, we performed a geometry optimization and reached the energy minimum, characterized by no imaginary frequencies (Figure 16). We performed a similar task for the product of the reaction, that is, after removing H^•^(4′) from quercetin, we posed its minimized radical at van der Waals separation from minimized HO_2_^−^ and the result of the corresponding geometry optimization is depicted in Figure 16. The transition state (TS) was found (Figure 16) and showed, as expected, only one imaginary frequency, −1128 cm^−1^. DeltaG for this calculation is −1.5 Kcal/mol and the associated barrier is 0.5 Kcal/mol, which indicates reaction feasibility.

**Figure 17:**
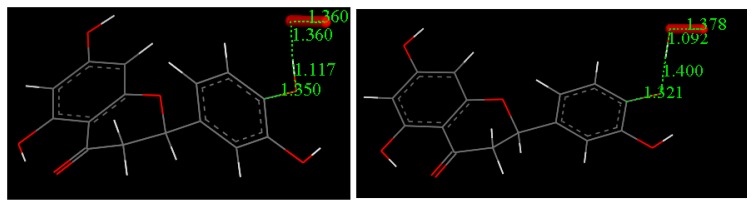
Minimized reagent, left, and product, right, for eriodictyol scavenging superoxide.

Eriodictyol coordinates were taken from the X-ray crystal structure presented here and used to run additional calculations for the equivalent superoxide scavenging (Figure 17); its DeltaG is −0.2 Kcal/mol. This small difference likely made obtaining the transition state difficult. Figure S1 shows that in the TS search energy profile, the highest part of the curve is of the same energy order as DeltaG, which means that the potential barrier is accessible. Our results agree with a CV study of catechin showing that the reaction of the catechol 3′−4′ dihydroxyl moiety occurs first, and is reversible [26]. The only difference between catechin and eriodictyol is an additional OH in position 3 of the pyrone (carbonyl-containing) (C) ring in the former.

These calculations show that the quercetin and eriodictyol can scavenge superoxide through eventual formation of semiquinone arrangements of the scavengers, supporting our experimentally found CV data. Chrysin has only 2 hydroxyls in A-ring position 5 and 7, and, due to the intramolecular H-bond established with the neighbor carbonyl, H5 is less reactive that H7, and so the latter was explored for scavenging superoxide. Therefore, an initial state for chrysin, similar to that for quercetin shown in Figure 15, was built to study the interaction between superoxide and chrysin. This involved the extraction of H^•^ in position 7, and geometry minimization was performed as above, and the resulting minimum is shown in Figure S2. We note there is a marked difference, however, when analyzing the product of reaction in comparing chrysin with quercetin. That is, after approaching HO_2_^−^ to the H7-excluded chrysin radical, the arrangement is identical to the reagent structure shown in Figure S2, therefore indicating no reactivity between chrysin and superoxide. Rene et al., have described an important mechanistic difference after applying classical CV to related compounds: when scavenging superoxide: phenols act differently than flavonoids, as, the latter transfer H^•^ to O_2_^•−^ radicals, whereas there is involvement of ion transfer for phenols [27]. Indeed, the only 2 hydroxyls of chrysin are located in a very different environment than the B ring in quercetin and we wondered if they could act like phenols as suggested by Rene. Therefore, we attempted to explore an alternative mode of scavenging suggested by the crystal structure of chrysin, which shows π-π interactions [28]. This involved using *two* molecules of chrysin, even though the flavonoid concentration used in the RRDE measurement is low, by posing a 2nd molecule of chrysin in π-π arrangement to the species depicted in Figure S3. We find a successful stabilization of the product, right view of Figure S3. Moreover, a calculated molecule of H_2_O_2_ shows d(O-O) of 1.447 Å, which is very similar to the related moiety in Figure S3, right. However, in this case, DeltaG is positive, 3 Kcal/mol, indicating no reactivity, yet, there is a stable product, and as Rene indicated, some complex ionic process may be suggested to explain the experimental scavenging of superoxide by chrysin as shown by CV in this work. In an additional attempt, we explored the interaction of superoxide, still with 2 molecules of chrysin, yet avoiding the π-π arrangement. Again, there is stabilization of the product and DeltaG is slightly smaller, 1.9 Kcal/mol, Figure S4. We conclude that the flavone chrysin acts differently from flavanol quercetin and/or flavanone eriodictyol, and through a more complex mechanism.

**Figure 18:**
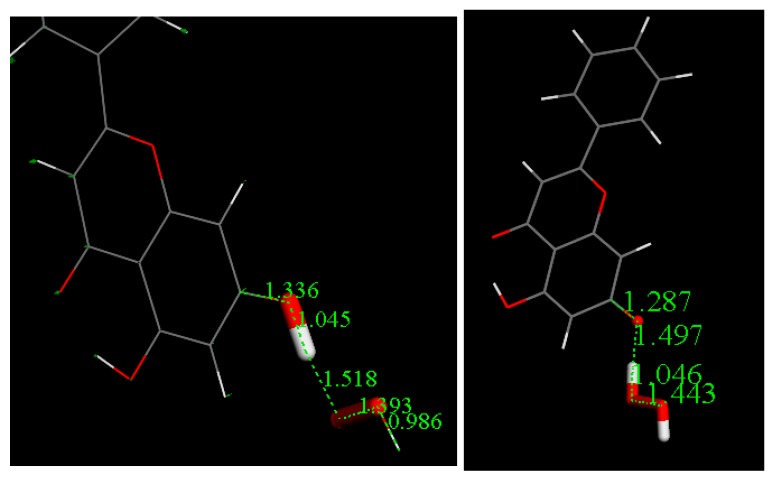
Final Reagent, left, and Product, right, after interaction by a hydronium on the O_2_ moiety shown in Figure S2, and after excluding a molecule of water.

Residual protons can also induce ionic interactions and so we posed the conformation shown in Figure S2 to the hydronium cation. Upon geometry minimization there was an important effect. The proton detaches from H_2_O and establishes a chemical bond to the O_2_ moiety, while the contact in the chrysin-superoxide moiety lengthens. The corresponding product, also geometrically optimized. Figure 18 shows the final situation with H_2_O excluded. The resulting DeltaG is negative, −1.0 Kcal/mol, suggesting reactivity. Therefore, our latter calculations indicate, theoretically, that chrysin reacts differently than quercetin and eriodictyol when scavenging superoxide.

## 5. Conclusions

In this work we selected 3 representatives of flavonoids, a flavone (chrysin), a flavanol (quercetin) and a flavanone (eriodictyol) with different number of hydroxyl groups to analyze their influence in the scavenging of superoxide radical. Voltammetry is well suited for monitoring redox reactions proceeding through electron transfer. We show the application of CV to produce, in situ, the reactive species, the superoxide radical ion, and then measure the amount of superoxide remaining a short time later. Moreover, the added antioxidant reacts directly with the CV generated superoxide allowing for fewer variables in the aprotic CV cell so that side reactions can be avoided. This method, then, is an attractive alternative to using a biological environment to generate superoxide. In addition, KO_2_ generation of superoxide generates instability, after titration of dry DMSO solutions. Through a calibration curve we can ascertain the actual concentration of superoxide ions and then its decrease of concentration due to added antioxidants.

As shown above, the classical CV method (Figure 7), detects decrease of current intensity of superoxide signal according to increasing concentration of flavonoids added to the electronic cell. In such a system both redox reactions take place at the same electrode [10]. However, there is not a marked difference in the CV response for closely related flavonoids.

An improvement is shown in the RRDE CV method, as the presence of an additional electrode (the ring electrode) allows for specific determination of the amount of superoxide remaining in solution after adding a given amount of antioxidant. This permits us to obtain more quantitative results regarding antioxidant scavenging ability, Figure 11 and Figure 13, for eriodictyol. The RRDE option is rapid, accurate, thorough and reproducible, suggesting an advantage over other methods used to measure superoxide scavenging in the literature [8]. In contrast with classical CV, when using the RRDE CV method, each hydroxyl group present in the three flavonoid molecules may be accounted for accurately, that is, the role of different flavonoids scavenging the superoxide radical can be well distinguished. This is observed for eriodictyol and chrysin whereas quercetin shows a more complex behavior with much higher scavenging activity and a nonlinear behavior of scavenging, in contrast with eriodictyol and chrysin. Indeed, the number and location of hydroxyl groups which determine how the resulting radical or radical ion can be stabilized, have long been considered as determining factors in antioxidant radical scavenging activity [9]. In fact, the hydroxyl substitution on the B ring appears to be important for antioxidant activity [10]. Accordingly, chrysin, having two OH substituents (none on the B ring), shows the lowest scavenging ability; quercetin with five OH groups, two of which are in a 3′,4′-catechol substitution pattern on B-ring, has the highest activity, while eriodictyol, with four hydroxyls, two of which are in a 3′,4′-catechol arrangement on B-ring shows intermediate scavenging ability.

Our DFT results show the superoxide scavenging by quercetin, eriodictyol and chrysin to be energetically and structurally feasible in agreement with the corresponding experimental CV data. However, mechanistic considerations from our DFT study suggest marked differences for scavenging superoxide by these three natural polyphenolic compounds, depending on whether hydroxyl groups are present (quercetin, eriodictyol) or missing (chrysin) in the B-ring.

The structure-function relationship for flavonoid protection of lipid peroxidation has been described [29]. The flavonoid pharmacophore involves a catechol moiety in ring B and a OH-group at the 3 position with electron donating groups at the 5 and/or 7 position in the AC-rings. Quercetin (with five hydroxyls at 3, 5, 7, 3′, 4′) is an example of a flavone having this molecular arrangement. This finding is in agreement with our experimental results where the quercetin superoxide scavenging is superior to the other two tested compounds. Eriodictyol (5, 7, 3′, 4′ OH and missing the 3-OH group) is next in scavenging potency and chrysin (5, 7 –OH groups) shows the least scavenging activity.

The limitations and side reactions of methods used for generation and analysis of superoxide have been recently reviewed [11]; however, no CV techniques were included. The growing field of electrochemical methods used to measure antioxidant activity, has also been reviewed recently. Some advantages with respect to traditional, more laborious instrumental techniques include: sensitivity, rapidity, simplicity, and no complicated sample pre-treatment [30]. We now add the possibility of using an enhanced electrochemical technique, the RRDE method, which permits the study, *unambiguously*, of the scavenging reaction at one electrode, and quantitatively, the superoxide consumed by antioxidants.

## Figures and Tables

**Table 1:** Crystal Data and Structure Refinement for Eriodictyol.

Empirical formula	C_15_ H_12_ O_6_
Formula weight	288.2
Temperature	125(2) K
Wavelength	0.71073 Å
Crystal system	Monoclinic
Space group	*Pc*
Unit cell dimensions	*a* = 16.650(3) Å
	*b* = 5.220(1) Å β = 90.894(3)°
	*c* = 14.217(3) Å
Volume	1220.4(4) Å^3^
Z	2
Density (calculated)	1.568 Mg/m^3^
Absorption coefficient	0.123 mm^−1^
F(000)	600
Crystal size	0.12 × 0.07 × 0.03 mm^3^
Theta range for data collection	2.84 to 25.03°
Index ranges	−19 ≤ h ≤ 19, −6 ≤ k ≤ 6, −16 ≤ l ≤ 16
Reflections collected	19484
Independent reflections	4304
Absorption correction	Empirical (SADABS)
Max. and min. transmission	0.9854 and 0.9963
Refinement method	Full-matrix least-squares on F^2^
Goodness-of-fit on F^2^	1.056
Final R indices [I > 2sigma(I)]	R^1^ = 0.0528, wR^2^ = 0.1053
R indices (all data)	R^1^ = 0.0988, wR^2^ = 0.1263

**Table 2:** Correlation data for antioxidant scavenging of superoxide. Slopes from the linear curves depicted in Figure 13 and Figure 14, and that calculated when including only data for low concentrated quercetin, e.g., left part of Figure 12.

Antioxidant	Number of OH Groups	Slope (1/M)	Ratio of Slopes
Quercetin	5	−5.30 × 10^4^	9.6
Eriodictyol	4	−2.20 × 10^4^	4
Chrysin	2	−1.10 × 10^4^	2

## Supplementary Materials

The following are available online at http://www.mdpi.com/2076-3921/8/1/14/s1.
