# Supplementary material for: Effective and Novel Application of Hydrodynamic Voltammetry to the Study of Superoxide Radical Scavenging by Natural Phenolic Antioxidants
Source: Antioxidants (Basel). 2019 Jan 4;8(1):14. doi: 10.3390/antiox8010014 (PMC6356364; doi:10.3390/antiox8010014)
Supplement: Supplementary file 1 [file antioxidants-08-00014-s001.pdf]

Deposited Figures

Figure S1 Transition State search for Eriodictyol scavenging of superoxide.

Figure S2 Minimum reached after approaching superoxide to chrysin H7. The same structure results after approaching HO_2^- to the H7-excluded chrysin radical.

Figure **S3** Conformation obtained after approaching the minimum shown in Figure **2** to an additional molecule of chrysin (Stick style), left. This π-π stacking arrangement is a radical of charge -1. The related minimized product, obtained after posing H_2O_2 at van der Waals separation from the 2 chrysin moieties, is displayed on the right.

Figure S4 Avoiding the π-π interaction in chrysin. Minimums obtained after geometry optimization of the species shown in Figure **S2**, line style, posed to another chrysin molecule (stick style), left, and its related product, right.

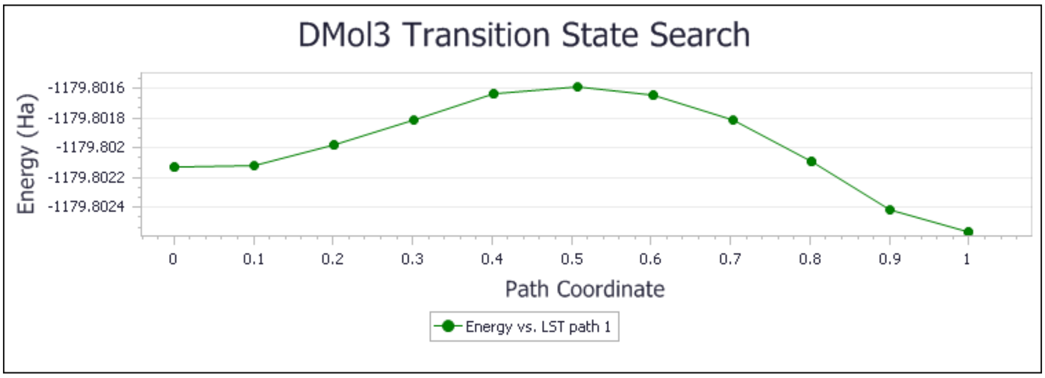

Figure S1

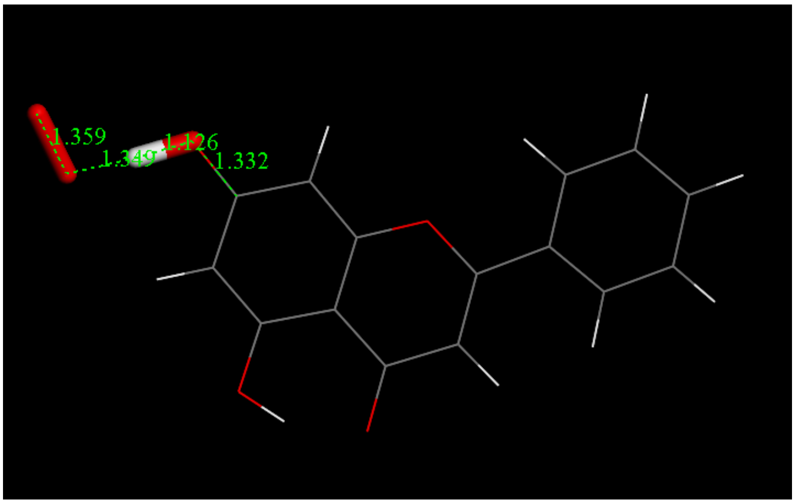

Figure S2

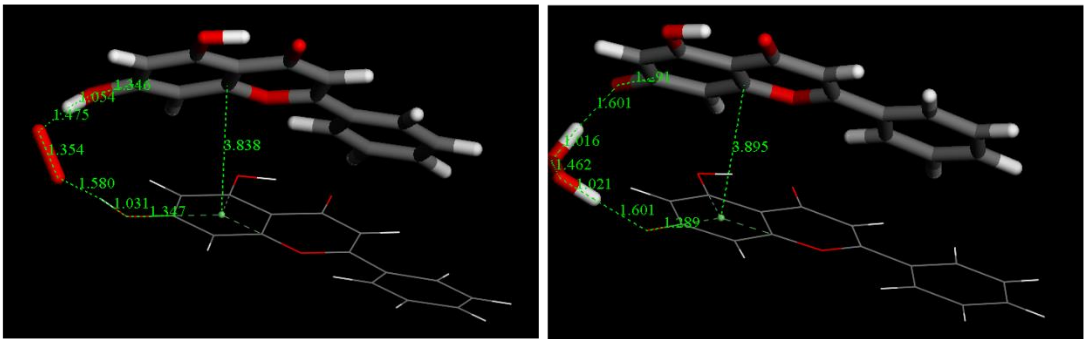

Figure S3

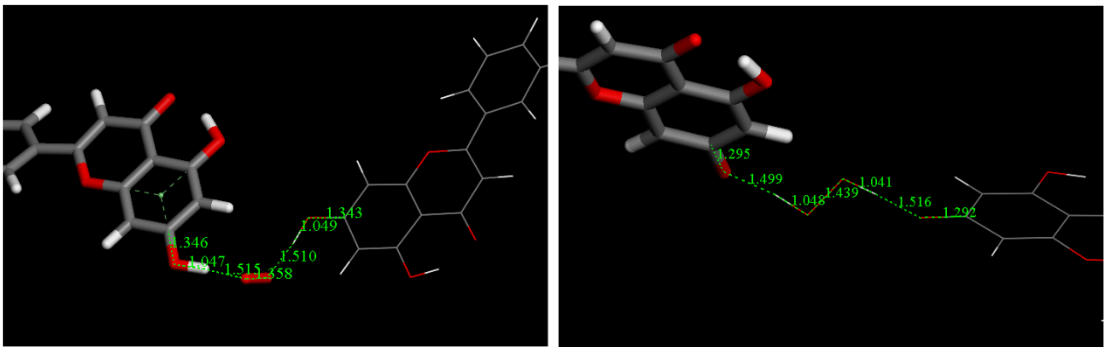

Figure S4
